# Wettability and friction control of a stainless steel surface by combining nanosecond laser texturing and adsorption of superhydrophobic nanosilica particles

**DOI:** 10.1038/s41598-018-25850-6

**Published:** 2018-05-10

**Authors:** M. Conradi, A. Drnovšek, P. Gregorčič

**Affiliations:** 1 Institute of metals and technology, Lepi pot 11, 1000 Ljubljana, Slovenia; 2 Jozef Stefan Institute, Jamova 39, 1000 Ljubljana, Slovenia; 3 Faculty of Mechanical Engineering, University of Ljubljana, Aškerčeva 6, 1000 Ljubljana, Slovenia

## Abstract

In this work, we present functionalization of AISI 316 L surfaces by nanosecond Nd:YAG laser texturing and adsorption of superhydrophobic fluoroalkylsilane functionalized 30-nm silica nanoparticles. Surface modification by varying the distance between laser-produced micro(μ)-channels leads to different surface roughnesses. After nanosilica coating, the superhydrophilic laser-textured surfaces change into superhydrophobic surfaces with the same μ-roughness. A higher μ-channel density leads to more hydrophobic surfaces after coating. This enables a study of the combined effect of surface wettability and morphology on the friction coefficient and wear resistance. Experiments were performed in dry and water environments. In the case of dry friction, increased μ-roughness leads to a higher friction coefficient, and the water-repellency modification by nanosilica particles has no influence on the tribological behaviour. In contrast, in the water environment, the wettability presents an important contribution to the properties of contact surfaces: hydrophobic surfaces exhibit a lower friction coefficient, especially at higher densities of μ-channels. Energy-dispersive X-ray spectroscopy analysis of surfaces before and after the tribological experiments is performed, revealing the difference in weight % of Si in the worn surface compared to the unworn surface, which varies according to the nature of the surface morphology due to laser texturing in both dry and water environments.

## Introduction

Manipulation and control of the surface properties of metallic substrates have attracted the interest of several researchers in the past few years from both basic research and application points of view. Several techniques, from organic/inorganic coating to surface texturing, have been reported to improve the functionalities of these substrates, including mechanical properties such as wettability, friction and wear behaviour, to extend the lifetime of mechanical systems^1–3^. Recently, laser surface texturing has been addressed as one of the most promising approaches for decreasing/increasing the friction coefficient under dry and lubricated conditions^4–8^. The most important advantages of laser surface engineering are its flexibility, accuracy, chemical-free production, lack of tool wear and negligible effect on the properties of bulk material^8^.

It has also been conveniently demonstrated that laser texturing enables flexible control of surface wettability from the superhydrophilic state in a saturated Wenzel regime (with a static contact angle of 0°) to superhydrophobic, lotus-leaf-like surfaces with contact angles exceeding 150° and sliding angles below 5° ^9–11^. In combination with appropriate surface chemistry, laser-textured surfaces exhibit promise as working material in different media. Superhydrophobic surfaces (i.e., coatings) are designed for protection against erosion in aqueous conditions, which represents a serious problem in terms of the loss of efficiency and performance in underwater operating systems^12^. Therefore, superhydrophobic coatings with good resistance to mechanical loads are significant in various industrial applications to obtain long-term performance. Many studies have been devoted to the development of durable superhydrophobic surfaces/coatings that present mechanical and anticorrosion barrier properties^12^. However, the synthesis of long-lived superhydrophobic coatings that retain their advantageous properties is still one of the important scientific challenges.

Most of the research in the fields of surface engineering for friction applications and wettability control addresses the isolated influence of surface modification on friction and wettability. Very little attention has, however, been paid to the combined effect of surface roughness and surface wettability - which can both be controlled by applying lasers - on the contact surface behaviour. Therefore, in this work, we report on the functionalization of stainless steel surfaces by combining direct laser texturing (DLT) and adsorption of superhydrophobic fluoroalkylsilane (FAS) silanated 30-nm nanosilica (SiO_2_) particles. This enables us to study the combined effect of surface roughness and wettability on tribological properties. Wear analysis is performed in dry and water environments, and a coefficient of friction is discussed for pure laser-textured (hydrophilic) surfaces in comparison to superhydrophobic nanosilica-coated laser-textured surfaces leading to superhydrophobic surfaces. Energy-dispersive X-ray spectroscopy (EDS) analysis of worn and unworn surfaces is discussed to extract the tribofilm properties in connection with the coefficient of friction.

## Results

### Surface morphology

**Figure 1:**
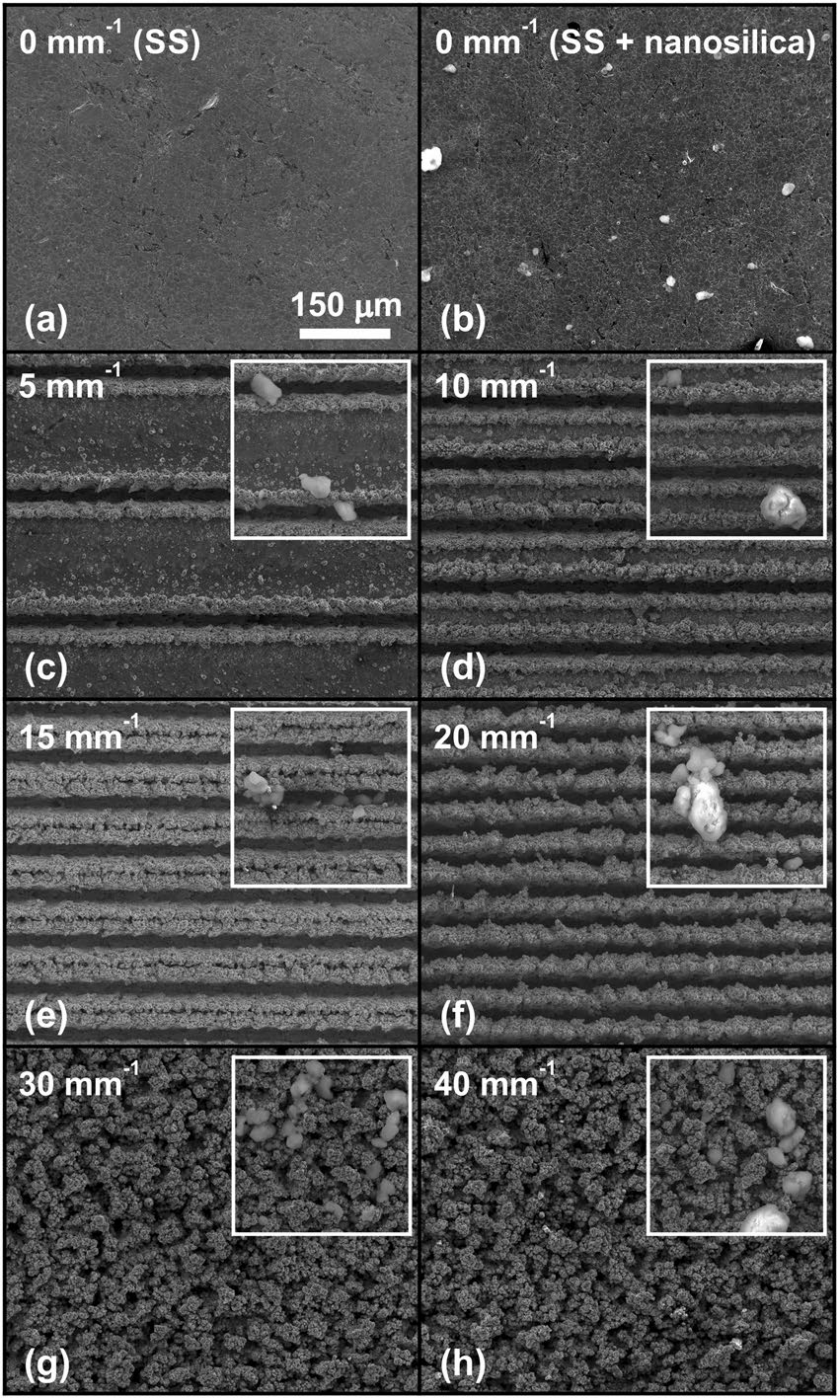
SEM micrographs of (**a**) an unprocessed surface (SS), (**b**) SS with 30-nm nanosilica particles and (**c**–**h**) laser-textured surfaces with different line densities Δy^−1^; the insets show the same surfaces after the adsorption of nanosilica particles. The scale is shown in (**a**) and it is the same for all the micrographs.

The as-received stainless steel surfaces were textured by a nanosecond marking laser with different line densities Δ*y*^−1^. The surface morphology depends on Δ*y*^−1^ (Fig. 1c–h) and defines the sliding environment. The unprocessed surface is shown as a reference in Fig. 1a. It is clear that the used fluences result in micro(μ)-channels. As already shown in ref.^11^, different line densities, *Δy*^−1^, lead to different surface morphologies. Here, high line densities (*Δy*^−1^ > 20 mm^−1^) result in a highly porous surface, with no distinguished μ-channels (Fig. 1g). In contrast, when the line density decreases, single μ-channels become visible, and the area of the unprocessed material between single μ-channels increases (Fig. 1c). After DLT, the surfaces were additionally coated via adsorption of fluoroalkylsilane (FAS)-functionalized 30-nm SiO_2_ nanoparticles, as shown in the insets in Fig. 1c–h. Here, nanosilica particle agglomerates with typical dimensions in the range of a few nanometres to a few tens of micrometres appear on the surface. Different sizes can be easily recognized from Fig. 1b, showing the nanosilica coating on unprocessed stainless steel. In the case of DLT surfaces, the nanosilica particles are mainly adsorbed by the “defects” induced by laser ablation (especially within and at the edges of μ-channels).

**Figure 2:**
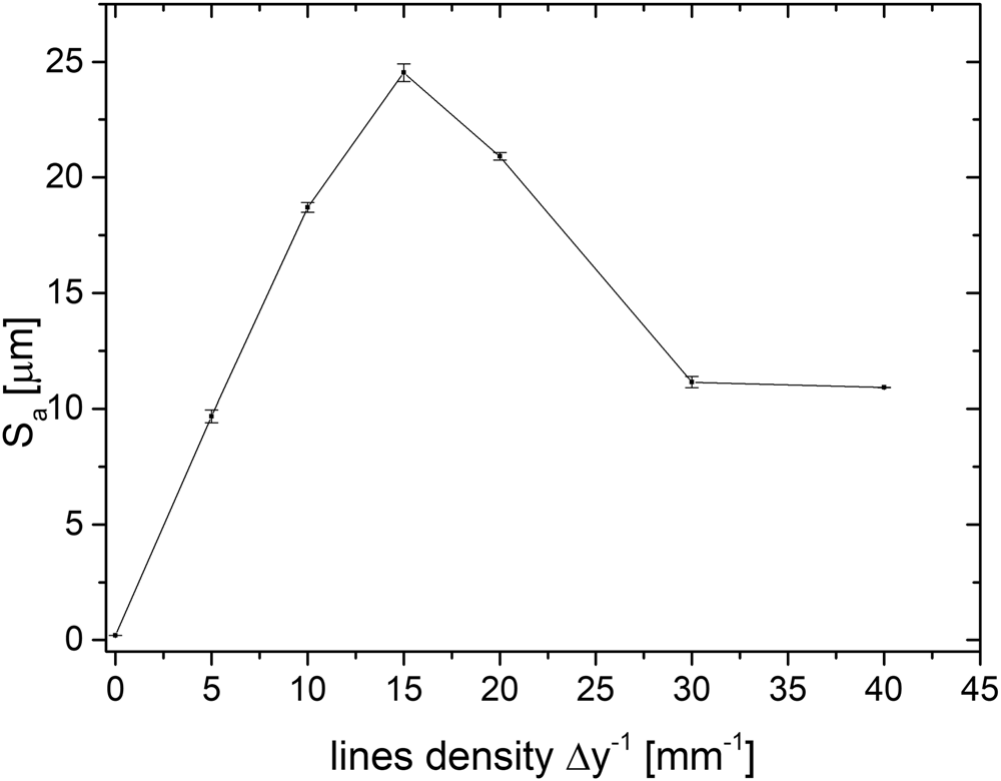
Change in the average surface roughness, S_a_, as a function of increasing line density *∆y*^−1^ on a laser-textured AISI 316 L surface.

The average surface roughness parameter, *S*_a_, was used as the main evaluation measure of the morphology difference between the laser-textured AISI 316 L surfaces. We performed five measurements on each surface over 1.3 × 1.0 mm^2^. As revealed in Fig. 2, the average surface roughness *S*_a_ increases with *Δy*^−1^ until 15 mm^−1^, where μ-channels are still well separated (see Fig. 1a–e). Here, the roughness increases from *S*_a_ = 202.7 ± 5.0 nm at *Δy*^−1^ = 0 (as-received unprocessed SS) to *S*_a_ = 24.5 ± 0.4 μm at *Δy*^−1^ = 15 mm^−1^. A further increase of *Δy*^−1^ leads to a decrease in the average surface roughness parameter because *S*_a_ measures the arithmetical mean of differences in the height of each point compared to the arithmetical mean of the surface. Therefore, it is not sensitive to the increased porosity induced by a further increase of Δ*y*^−1^ (see Fig. 1f–h).

### Surface wettability

To analyse the surface wettability, we performed 5 static water contact angle measurements, *θ*, with 5 μL droplets of deionized water on different spots all over the sample and used them to determine the average contact angle values with an estimated error in the reading of *θ* ± 2.0°. The unprocessed as-received surface (with *S*_a_ = 202.7 ± 5.0 nm) has a contact angle of *θ* = 80.0 ± 2.0°.

**Figure 3:**
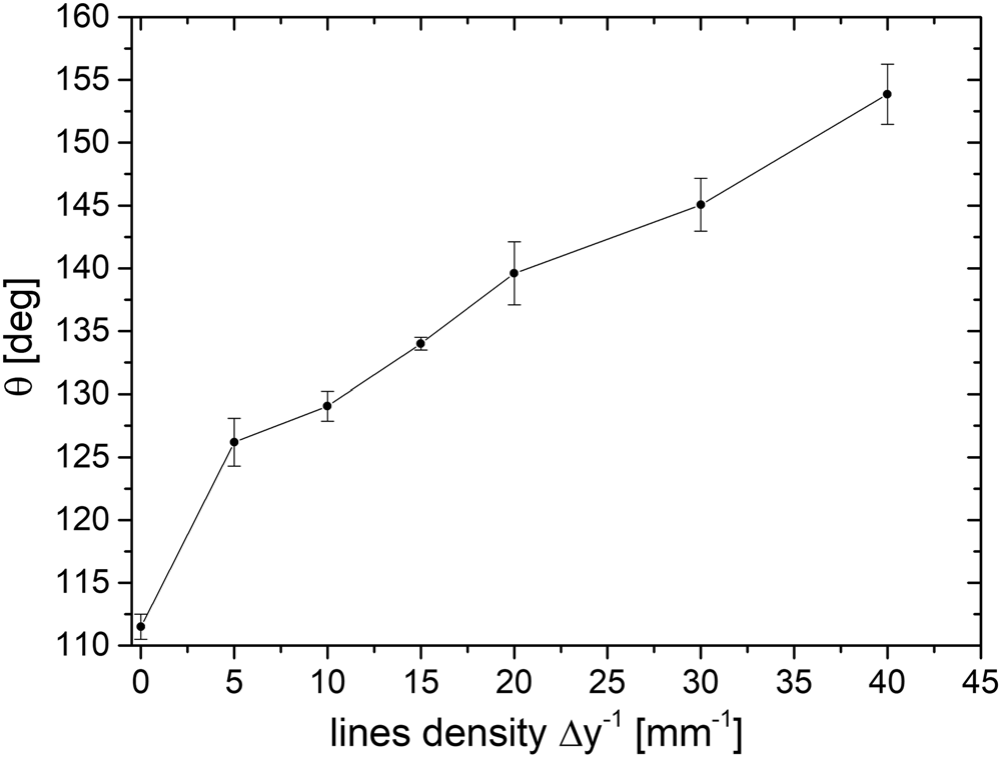
Change in the static water contact angle *θ* as a function of increasing line density *∆y*^−1^ on a laser-textured AISI 316 L surface functionalized with 30-nm FAS-SiO_2_ nanoparticles.

After DLT, all surfaces are superhydrophilic in a saturated Wenzel regime^13^ with a static water contact angle of 0°, as previously explained in ref.^11^. However, the adsorption of FAS-functionalized nanosilica particles changes superhydrophilic DLT surfaces into a (super)hydrophobic state. Here, the hydrophobicity strongly depends on the line density; higher *Δy*^−1^ leads to a higher contact angle, as shown in Fig. 3. A superhydrophobic surface with a contact angle of *θ* = 153.8 ± 2.4° is obtained for *Δy*^−1^ = 40 mm^−1^. In the case of an unprocessed surface (*Δy*^−1^ = 0) coated with nanosilica particles, the contact angle equals 111.5 ± 1.0°.

### Tribological behaviour

**Figure 4:**
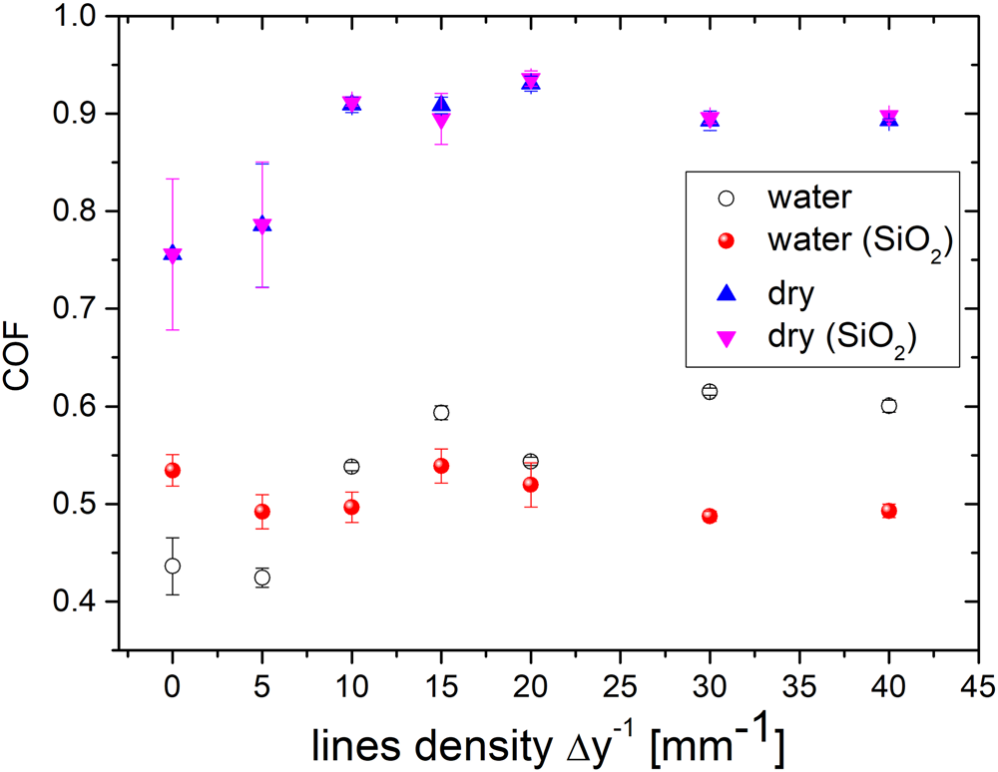
Friction coefficient (COF) in dry and water environments as a function of increasing line density *∆y*^−1^ on a laser-textured AISI 316 L without and with 30-nm FAS-SiO_2_ nanoparticles.

The tribological properties of functionalized contact surfaces in dry and water environments were tested in a single-way point contact setup with an alumina ball as a counter body material; here, a 5 mm radius with a 5 N normal load was used. To distinguish between the influence of surface morphology (roughness) and wettability, we tested superhydrophilic DLT surfaces and DLT surfaces with the same μ-roughness that became hydrophobic due to FAS-nanosilica coating. The measurement of the coefficient of friction (COF) as a function of *Δy*^−1^ in Fig. 4 reveals that the wettability does not play any role in the dry environment; instead, the friction coefficient increases with *Δy*^−1^ and reaches a saturated value when μ-channels begin to overlap (at *Δy*^−1^ > 20 mm^−1^). Here, the lowest COF is obtained for the unprocessed surface; in this case, it equals ~0.7, and it increases up to 0.9 with increasing *Δy*^−1^ (the blue triangles in Fig. 4). Additional functionalization by FAS-nanosilica particles resulting in more water-repellent surfaces has little effect on the COF (the pink triangles in Fig. 4).

**Figure 5:**
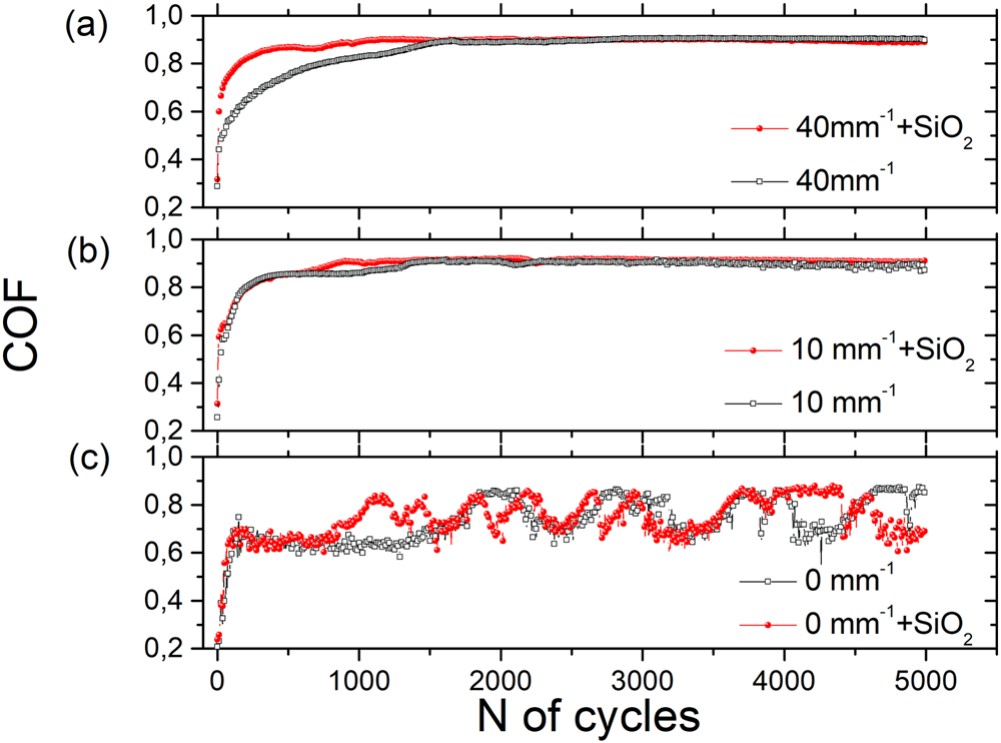
Typical COF lines for laser-textured AISI 316 L surfaces without and with 30-nm FAS-SiO_2_ nanoparticles in a dry environment. A clear difference in the running-in behaviour after adding nanoparticles is visible for samples with high *Δy*^*−1*^ (**a**), and no difference is observed for samples with low *Δy*^−1^ (**b**). For unprocessed samples (**c**), oscillations in the COF appear.

A minor difference appears only in the COF during the running-in period (Fig. 5a and Supplementary Fig. 1) for samples with high line density, *Δy*^−1^ = 40 mm^−1^. During this period, the COF rapidly increases as the wear particles are generated and more conformal contact between sliding bodies is formed. Because of pre-existing FAS-SiO_2_ nanoparticles on the nanosilica-coated surfaces, this running-in period is shorter compared to textured surfaces without FAS-SiO_2_. This is, however, not noted in samples with lower line density, *Δy*^−1^ = 10 mm^−1^ (Fig. 5b). The COF of unprocessed samples exhibited friction oscillations after 1000 cycles: when FAS-SiO_2_ nanoparticles were added, this value increased to 1500 cycles (Fig. 5c).

The situation is significantly different in the water environment (Supplementary Fig. 2). Here, the lowest COF is again obtained on the as-received unprocessed SS and equals ~0.4. In the case of DLT (superhydrophilic) surfaces, the COF also increases with *Δy*^−1^ (the black points in Fig. 4) and reaches a saturated value of approximately 0.6. Therefore, the COF in the water environment is shifted by ~−0.3 compared to the dry environment because water acts as a lubricant. However, in a water environment, not only the surface roughness but also the surface wettability plays a crucial role. Water leads to the splitting effect – hydrophilic surfaces experience different COFs than hydrophobic surfaces with the same μ-roughness when tested in water. In the case of hydrophobic surfaces (the red dots in Fig. 4), the roughness does not play a significant role because the dependence of the measured COF on *Δy*^−1^ is not significant. The interesting result is that hydrophobic surfaces with lower roughness (*Δy*^−1^ < 10 mm^−1^) result in higher COF values than those for hydrophilic surfaces and vice versa for *Δy*^−1^ ≥ 10 mm^−1^. The largest difference in COFs between hydrophilic and hydrophobic surfaces in the water environment was observed for samples with line density *Δy*^−1^ ≥ 30 mm^−1^ due to the maximal wetting difference between the two surfaces, superhydrophilic vs. superhydrophobic.

**Figure 6:**
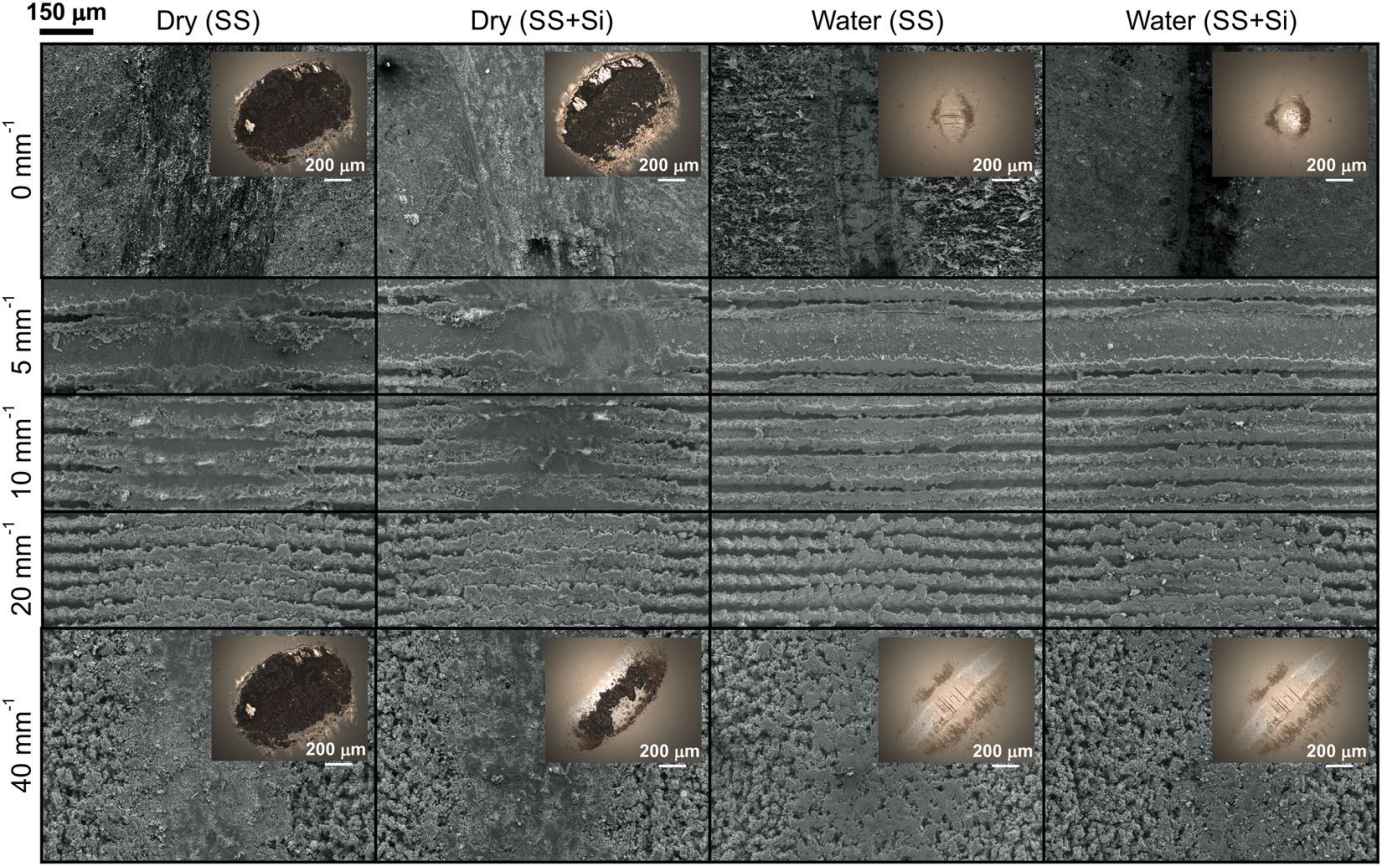
SEM images of selected wear tracks obtained in dry and water environments. The insets in the images show the worn surface of the sliding ball.

Wear of the surfaces was evaluated by SEM imaging after the tribological testing (Fig. 6 and Supplementary Fig. 2). High friction in the dry environment resulted in higher wear for all samples compared to wear in the water environment. The µ-channels on the textured samples are smeared, and the spacing between them is filled with wear debris, producing a smoother wear track surface than the unworn surface. In general, the FAS-SiO_2_ nanoparticles are not lubricous, nor do they have anti-wear properties. Therefore, their role in dry-wear behaviour is not significant. In contrast, the wear in the water environment is severely reduced, and only the peaks and edges of µ-channels are affected by the sliding counter body. The wear track is, however, still interrupted by the µ-channels.

Independent of the sliding environment, dry or water, the wear tracks are wider on laser-textured surfaces compared to unprocessed AISI 316 L samples. Nevertheless, this difference is more pronounced in the water environment.

The counter body wear (Supplementary Fig. 4), due to the high hardness of alumina, is minimal. The wear is more uniform when sliding against unprocessed samples, and more material is simultaneously transferred to the counter body in this case (dark material on the ball). Sliding against textured samples, on the other hand, causes more uneven wear of the ball. This results in a wide elliptic wear scar on the counter body. FAS-SiO_2_ nanoparticles do not significantly influence counter body wear. In contrast, the change of the sliding environment to water completely eliminates the galling of the wear material on the counter body.

**Figure 7:**
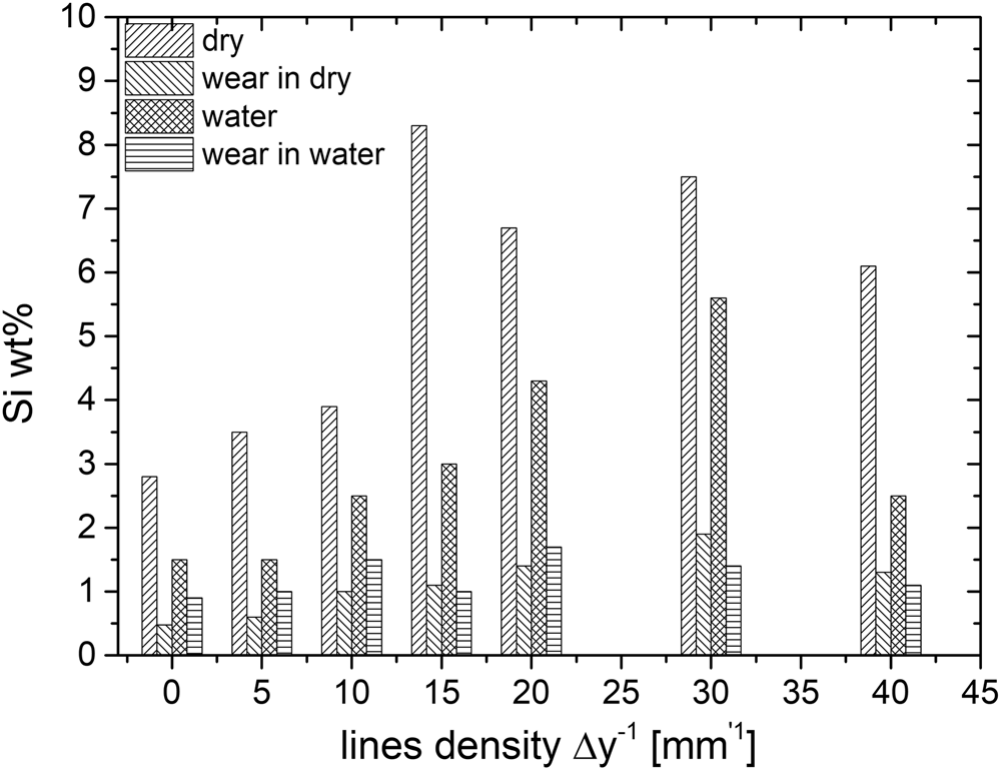
Si wt% before and after wear analysis in dry and water environments for unprocessed and laser-textured surfaces as determined by EDS analysis.

EDS analysis, presented in Fig. 7, reveals the difference in weight % of Si in the worn surface compared to the unworn surface, which also varies according to the nature of the surface morphology due to laser texturing. In the unworn surface (before the tribological experiments), the Si wt% increases with increasing *Δy*^−1^ and saturates at the maximal value for the laser-textured surface with line density above *Δy*^−1^ ≥ 20 mm^−1^. Due to the overlapping of the laser lines, these textures become inseparable (Fig. 1), offering much space for silica nanoparticle trapping. The Si wt% follows the same trend in dry and water environments; only the amount of Si is lower in water, most likely due to the water flow that washes away excess material.

The Si wt% for a given line density in worn surfaces (after the tribological experiments) is smaller in the dry compared to the water environment for *Δy*^−1^ < 15 mm^−1^. For larger values of *Δy*^−1^, the difference in Si wt% in both environments is no longer significant.

## Discussion

The friction coefficient in a dry environment is high. It spans from 0.7 to 0.9 for different line densities. Samples with small line density (i.e., *Δy*^*−1*^ = 5 mm^−1^) have smaller roughness (S_a_) and low COF in dry conditions. In this case, the mechanical interlocking of smooth surfaces is low; therefore, such surfaces exhibit lower friction^14,15^. In addition, for the smallest line density (*Δy*^*−1*^ = 5 mm^−1^), the μ-channels are widely separated, and sliding with a relatively large ball (6 mm diameter) produces a very small increase in the friction coefficient.

In addition, the µ-channels act as microtraps for (abrasive) debris generated during sliding^16,17^. Therefore, at low line densities, their ability to remove the wear debris from the tribological contact is reduced. The µ-channels are quickly filled, and the edges are smeared out, which results in a levelled worn surface. The debris can then gather in front of the counter body, which increases the friction until the debris is removed. This may be a reason for oscillations appearing in the COF behaviour^18^. Such a phenomenon is not observed in samples with line density *Δy*^*−1*^ ≥ 10 mm^−1^ (Supplementary Fig. 1) because frequent gaps between laser lines prohibit the material from sticking on the counter body or gathering in front of it^19^.

The COF gradually increases with increasing line density, reaching a saturated value of approximately 0.9 for samples with line density above 10 mm^−1^. The maximum COF is measured on the samples near the peak surface roughness (*S*_a_). With increasing line density, the 30- and 40-mm^−1^ samples morphologically change from µ-channelled structures to highly porous surfaces with many μ-cavities (Fig. 1). Although the surface roughness decreases severely for the latter samples, there is no change in the COF towards lower values.

The addition of FAS-SiO_2_ nanoparticles to the sample surface does not have an influence on the average COF in a dry environment. The FAS-SiO_2_ nanoparticles do not offer any anti-friction or anti-wear protection properties, in contrast to solid lubricants such as MoS_2_^20^. However, the surface texturing role is similar to that with the use of solid lubricants; it increases the amount of FAS-SiO_2_ on the surface and in contact with the ball and therefore enables a constant feed (flow) of FAS-SiO_2_ to the tribological contact during operation^20^. Textured surfaces without solid lubrication have worse tribological behaviour compared to smooth surfaces in dry environments, as shown by the triangles in Fig. 4^16^. When lubrication is poor, the main effect of µ-texturing is likely to be the ability to remove wear particles from the sliding interface, i.e., filling the µ-channels with wear debris^16,21^.

The COF for all samples is lowered when water is introduced. Water in the tribological contact acts as a lubricant, i.e., it lowers the friction coefficient. Nevertheless, the COF trend is similar to that in the dry environment (Fig. 4), meaning that the surface geometry and morphology contributions to the COF remain the same. Consequently, due to the lower COF in water, the wear at µ-channel edges is less pronounced in water, than under sliding in a dry environment (Fig. 6).

However, dissimilarities in the friction behaviour in the water environment arise between the samples with and without FAS-SiO_2_ nanoparticles (Fig. 4). The hydrophobic nature of FAS-SiO_2_ nanoparticles becomes significant in the water environment, leading to different COF trends on surfaces coated with hydrophobic FAS-SiO_2_ nanoparticles. All the tested FAS-SiO_2_ samples exhibit similar COF values. As a function of line density, COF spans from 0.48 to 0.53. The maximal difference in COFs between the laser-textured surfaces coated with FAS-SiO_2_ nanoparticles and the superhydrophilic laser-textured surface without particles was observed at *Δy*^*−1*^ = 30 mm^−1^ (Fig. 4), where the surfaces become superhydrophobic after silica coating (Fig. 3). The COF of superhydrophobic surfaces is the lowest, making the surface the most resistant to abrasion wear in aquatic media. In contrast, the COF of the FAS-SiO_2_-coated laser-textured surface becomes higher in water compared to the COF of the laser-textured surface with no particles when *Δy*^*−1*^ ≤ 10 mm^−1^ (Fig. 4). Samples with *Δy*^*−1*^ = 10 mm^−1^ and 20 mm^−1^ experience a gradual increase of the COF towards the COF value of samples without FAS-SiO_2_ nanoparticles during the tribological tests (Supplementary Fig. 2). This could indicate both a gradual wear of the FAS-SiO_2_ coating and the loss of the superhydrophobic effect. Such behaviour was not noted for samples with *Δy*^*−1*^ = 30 mm^−1^ and 40 mm^−1^. However, it resulted in a long-maintained superhydrophobic effect that did not diminish through the length of our tests, suggesting the importance of both the micro-laser-textured morphology and nanosilica structuring in synthesizing a surface with desirable abrasion wear resistant properties that can be used in aquatic media for various applications.

Finally, the tests described in this manuscript were designed to observe the effect of FAS-SiO_2_ nanoparticles on the tribological behaviour when the wear of the counter body is minimal. The tribological behaviour would most certainly change with the use of softer but more realistic counter body material, such as steel instead of alumina. High wear of soft counter body materials usually leads to galling (sticking) of the material to the sample surface. This could lead to covering of the FAS-SiO_2_ coating and conversion of the surface back to (super)hydrophilic.

## Conclusions

We present a simple method for converting a superhydrophilic laser-textured AISI 316 L surface into a (super)hydrophobic surface by coating with FAS-SiO_2_ nanoparticles. Control of the wettability by varying the surface roughness via direct laser texturing of differently separated μ-channels enabled us to extract a friction coefficient and wear resistance in dry and water environments as a result of the combined effect of surface wettability and morphology. We have shown that in a dry environment, the COF is high and gradually increases with line density until reaching a saturated value, which is not affected by the morphology and the change in the surface roughness. FAS-SiO_2_ coating does not offer any anti-friction or anti-wear protection properties, which is reflected in the lack of influence on the average COF in a dry environment. In the water environment, the COF behaviour of superhydrophilic laser-textured samples follows a similar trend with the line density as in the dry environment, only at lower values due to water lubrication. For FAS-SiO_2_-coated surfaces, the COF trend is, however, changed, and the COF is significantly reduced when the coated laser-texture surface becomes superhydrophobic. This is also reflected in the increased wear resistance, where only the peaks and edges of μ-channels are affected by the sliding counter body. Superhydrophobic FAS-SiO_2_-coated laser-textured samples, therefore, offer an excellent wear resistant platform with long-lasting superhydrophobicity that did not diminish through the length of our tests for various applications in demanding working conditions in aquatic media.

## Methods

### Materials

Austenitic stainless steel AISI 316 L (17% Cr, 10% Ni, 2.1% Mo, 1.4% Mn, 0.38% Si, 0.041% P, 0.021% C, and <0.005% S in mass fraction) was used as a substrate. The AISI 316 L steel sheet with a thickness of 1.5 mm was cut into discs of 15 mm diameter. Silica (SiO_2_) nanoparticles with mean diameters of 30 nm were provided by Cab-O-Sil.

### Laser surface irradiation

All surfaces were direct laser textured (DLT) using a nanosecond Nd:YAG laser (wavelength of 1064 nm) with a pulse duration of 95 ns (full width at half maximum) in a similar way as in ref.^11^. The surfaces were textured by directing the laser beam along parallel lines with a velocity of *v* = 1.*6* mm s^−1^. We processed different surfaces with the following scanning line separations: *Δ*y = {25, 33, 50, 67, 100, 200} μm; therefore, the scanning line densities equalled *Δ*y^−1^ = {40, 30, 20, 15, 10, 5} mm^−1^. In all cases, we used the same pulse repetition rate *f* = 1 kHz and average power *P* = 0.6 W; consequently, the pulse energy equalled 0.6 mJ. The surfaces were placed in a focal position of an F-theta lens (focal length of 160 mm), where the beam waist diameter equalled 0.05 mm. The peak fluence, therefore, equalled *F*_0_ = 31 J cm^−2^, while the threshold fluence for laser ablation was estimated to be 7 J cm^−2^ (by decreasing the fluence to the value at which the laser did not induce morphological surface modification).

### Silica coating

For hydrophobic effects, SiO_2_ nanoparticles were functionalized in 1 vol% ethanolic fluoroalkylsilane or FAS (C_16_H_19_F_17_O_3_Si, Sigma-Aldrich) solution. FAS-functionalized 30-nm SiO_2_ nanoparticles were adsorbed onto a laser-patterned AISI 316 L surface by spin-coating 20 μl of 3 wt% SiO_2_ nanoparticle ethanolic solution.

### Tribological testing

Tribological testing was performed with a standard room temperature CSM (now Anton Paar) tribometer. We used the single-way mode in the ball-on-disc configuration (point contact). For the counter body, we used a 6-mm diameter Al_2_O_3_ ball (≈2000 HV) due to its high hardness and inertness. The experiments were conducted at a 5 mm radius with a normal load of 5 N. This corresponds to a maximal Hertz pressure of 1.2 GPa at the contact, calculated for a flat surface. Tests 5000 cycles in length (157 m) were performed in ambient laboratory conditions (22 °C, 40% RH) and in deionized water, which was replaced for each measurement to avoid contamination with silica particles. Prior to the individual test, the sample was purged with nitrogen gas to remove the presence of dust particles.

### Surface characterization

The morphologies of the DLT surfaces as well as DLT surfaces coated with FAS-SiO_2_ nanoparticles before and after the tribological tests were characterized by a JEOL JSM-6500F field emission scanning electron microscope (SEM) with attached energy dispersive X-ray spectroscopy (EDS; INCA ENERGY 400).

The surface wettabilities of the laser patterned surface and laser patterned surface coated with FAS-SiO_2_ nanoparticles were qualified with static water contact angle measurements of 5 μL deionized water droplets deposited on five different spots of the substrates to avoid the influence of roughness and gravity on the shape of the droplet. A surface energy evaluation system (Advex Instruments s.r.o.) was employed to analyse the droplets and measure contact angles.

An optical 3D metrology system, model Alicona Infinite Focus (Alicona Imaging GmbH), and IF-MeasureSuite (Version 5.1) software were employed for the surface roughness characterization. At least three measurements per sample were performed at magnification 20× with a lateral resolution of 0.9 μm and a vertical resolution of approximately 50 nm on an area of 1.337 × 0.993 mm^2^. The average surface roughness, S_a_, was evaluated from the general surface roughness equation (eq. 1):

$$Sa=\frac{1}{{L}_{x}}\frac{1}{{L}_{y}}{\int }_{0}^{{L}_{x}}{\int }_{0}^{Ly}|z(x,y)|dxdy,$$

where *L*_*x*_ and *L*_*y*_ are the acquisition lengths of the surface in the *x* and *y* directions and *z*(*x*, *y*) is the height.

### Data availability

The datasets generated and/or analysed during the current study are available from the corresponding author upon reasonable request.

## Electronic supplementary material

Supplementary Information
