# Supplementary material for: Wettability and friction control of a stainless steel surface by combining nanosecond laser texturing and adsorption of superhydrophobic nanosilica particles
Source: Sci Rep. 2018 May 10;8:7457. doi: 10.1038/s41598-018-25850-6 (PMC5945667; doi:10.1038/s41598-018-25850-6)
Supplement: Supplementary file 1 — Supplementary Information [file 41598_2018_25850_MOESM1_ESM.docx]

Supplementary Information

**Wettability and friction control of stainlesssteel surface by combining nanosecond-laser texturing and adsorption of superhydrophobic nanosilica particles**

**M. Conradi^1*^, A. Drnovšek^2^, and P. Gregorčič^3^**

^1^Institute of metals and technology, Lepi pot 11, 1000 Ljubljana, Slovenia

^2^Jozef Stefan Institute, Jamova 39, 1000 Ljubljana, Slovenia

^3^Faculty of Mechanical Engineering, University of Ljubljana, Aškerčeva 6, 1000 Ljubljana, Slovenia

**Supplementary Figures**


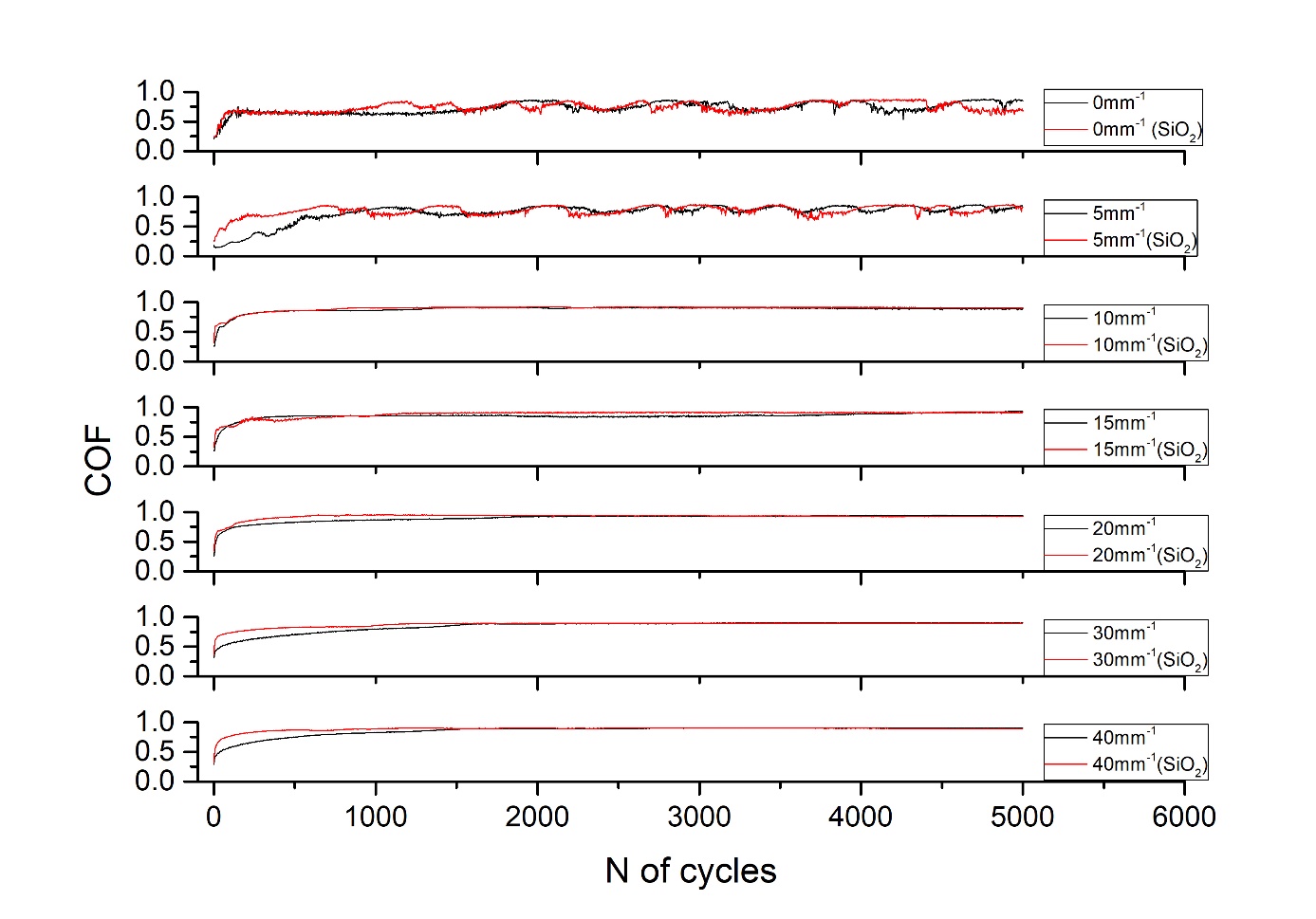


**Supplementary Figure 1:** Typical COF lines for laser-textured AISI 316L surfaces without and with FAS-SiO_2_ nanoparticles in dry environment for all lines densities under investigation. Clear difference in running-in behaviour after adding nanoparticles is visible for samples with high *Δy^-1^* and no difference is observed for samples with low *Δy^-1^*. For non-processed samples and for samples with *Δy^-1^* = 5 mm^-1^ the oscillations in COF appear.


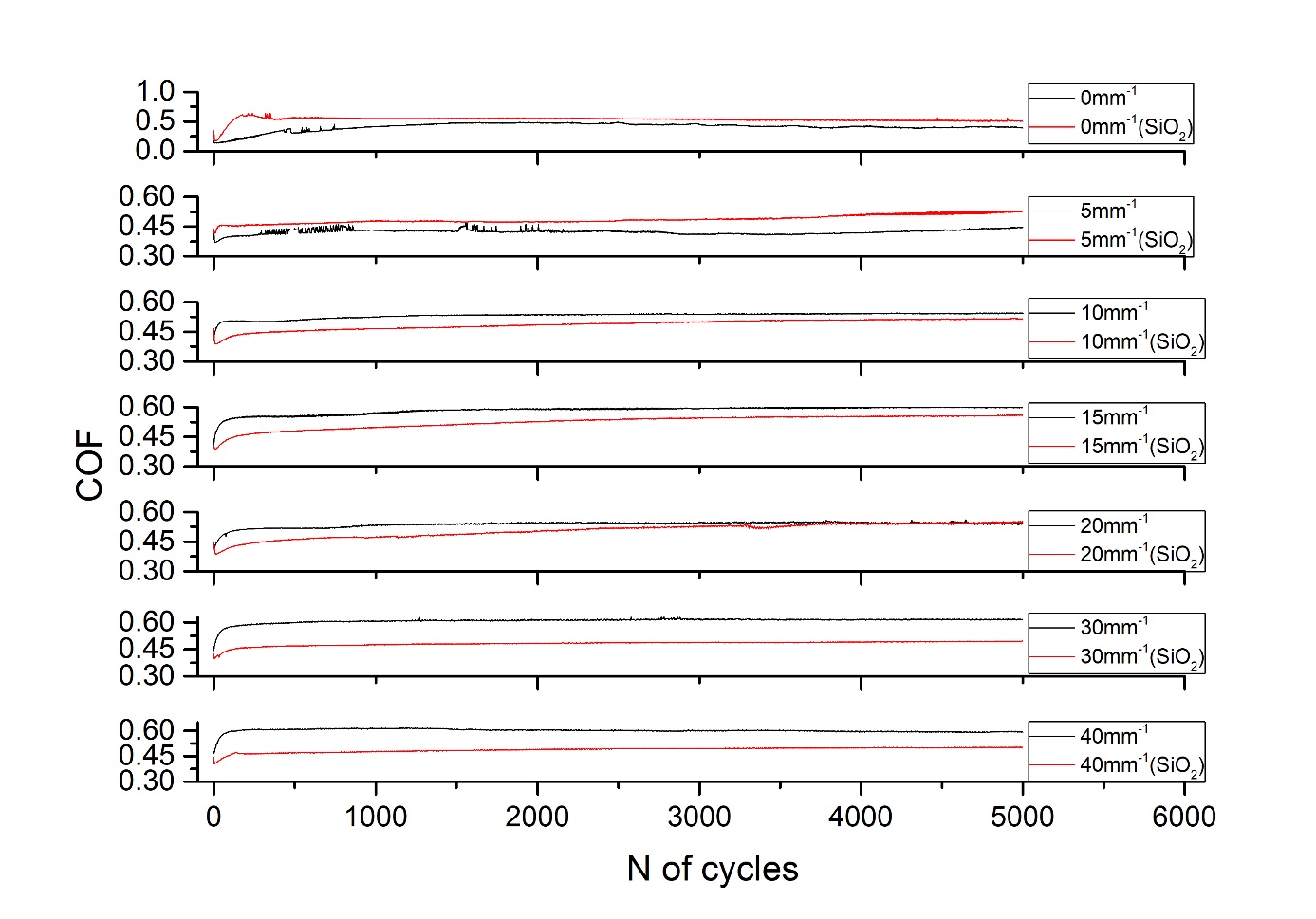


**Supplementary Figure 2:** Typical COF lines for laser-textured AISI 316L surfaces without and with FAS-SiO_2_ nanoparticles in water environment for all lines densities under investigation. Clear difference in running-in behaviour compared to experiments in dry environment is observed for unprocessed samples and samples with *Δy^-1^* = 5 mm^-1^. This is most probably due to the fact that water washed away the pre-existing silica nanoparticles. In addition, COF of silica coated samples with *Δy^-1^* ≥ 10 mm^-1^ is smaller compared to samples without nanoparticles.


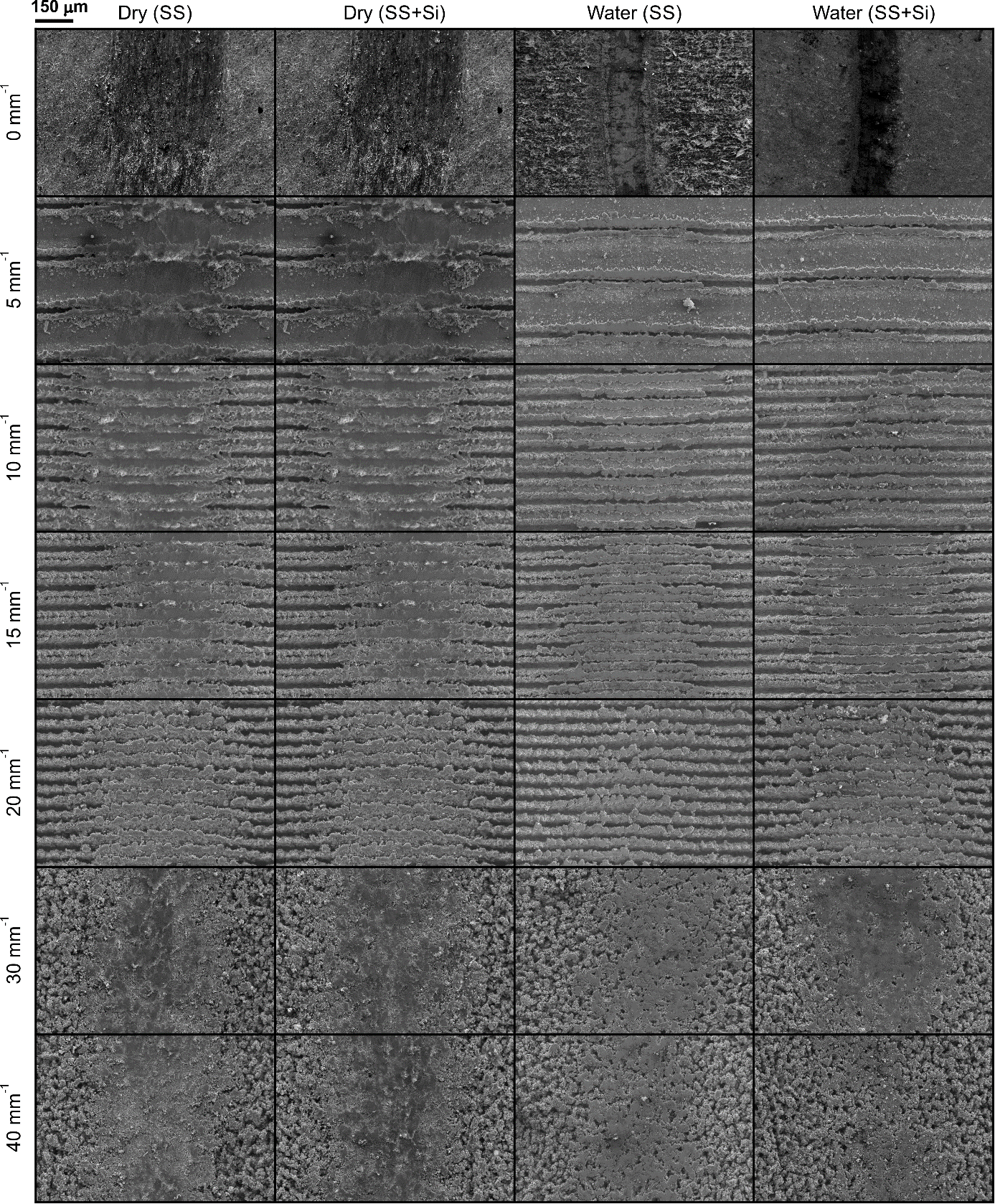


**Supplementary Figure 3:** Secondary electron images of wear tracks obtained in dry and water environment for all scan line separations.


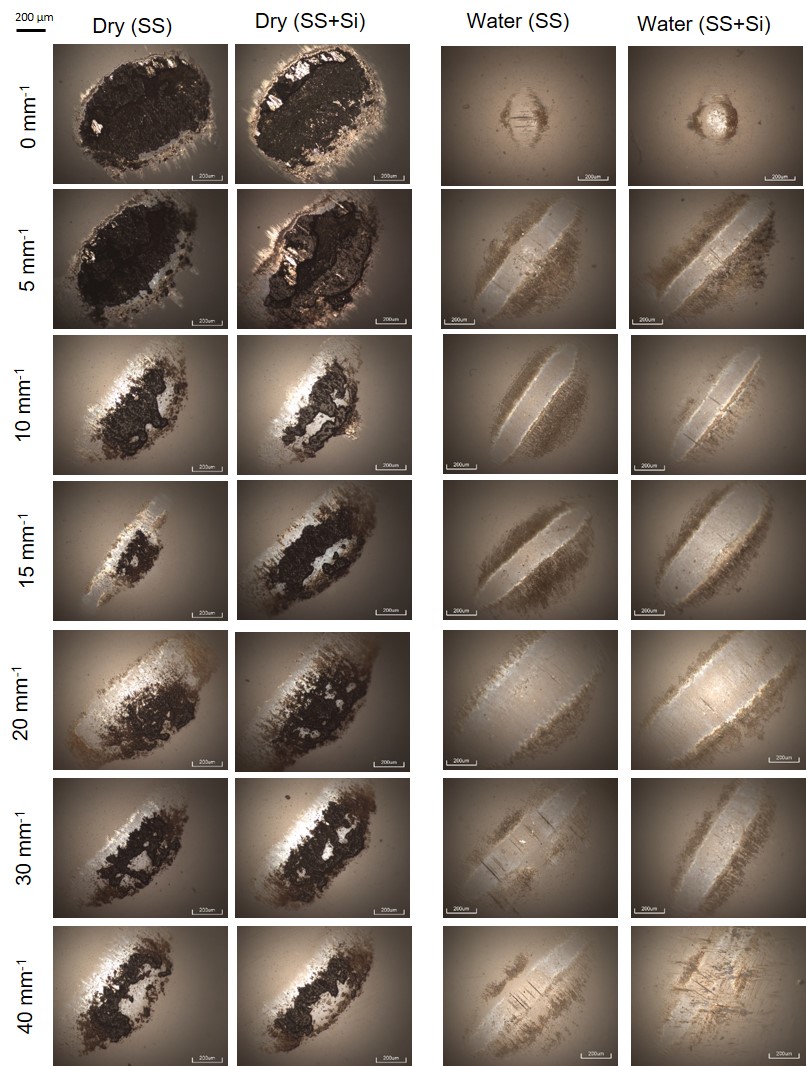


Supplementary Figure 4: The worn surfaces of the counter body – sliding alumina ball in dry and water environment on the surfaces for all scan line separations.
